# Systemic sclerosis: key clinical features of a rare and progressive disease

**DOI:** 10.11604/pamj.2025.51.7.47189

**Published:** 2025-05-08

**Authors:** Anshu Tikariha

**Affiliations:** 1 Department of Neuro Physiotherapy, Ravi Nair Physiotherapy College, Datta Meghe Institute of Higher Education and Research, Wardha, Maharashtra, India

**Keywords:** Systemic sclerosis, acro-osteolysis, physiotherapy

## Image in medicine

Systemic sclerosis (SSc) is a rare connective tissue disorder. Ischemia may lead to ulcerations and, in severe cases, digital resorption. A 53-year-old Indian female presented to the physiotherapy department with a primary complaint of neck pain numerical pain rating scale (NPRS) 5 during shoulder elevation, along with difficulty in hand function and mouth opening. She was diagnosed with SSc 20 years ago and was on regular pharmacological treatment. On observation, as depicted in (A), she had a mask-like face with microstomia and prominent radial furrowing, characteristic of systemic sclerosis. Panel B illustrates both hands with acro-osteolysis, sclerodactyly, onycholysis, digital ulcers, and multiple finger flexion contractures. On examination, the shoulder range of motion (ROM) was normal. Still, mild tightness was noted in the trapezius, levator scapulae, and pectoralis major, along with marked weakness of the serratus anterior manual muscle testing (MMT) grade 2/5. There was a significant restriction in both active range of motion (AROM) and passive range of motion (PROM) wrist and finger mobility, with a complete loss of precision grips. Mouth opening was moderately restricted (grade 2 limitation). A home exercise program was prescribed, including hot water fomentation, self-stretching, strengthening, cervical stabilization, hand and facial exercises. After four weeks, neck pain was relieved entirely (NPRS 0), with minimal improvement in active finger mobility and unchanged mouth opening. This case highlights the characteristic musculoskeletal manifestations of SSc, emphasizing the importance of physiotherapy and regular follow-ups in maintaining function and delaying disability in this progressive condition.

**Figure 1:**
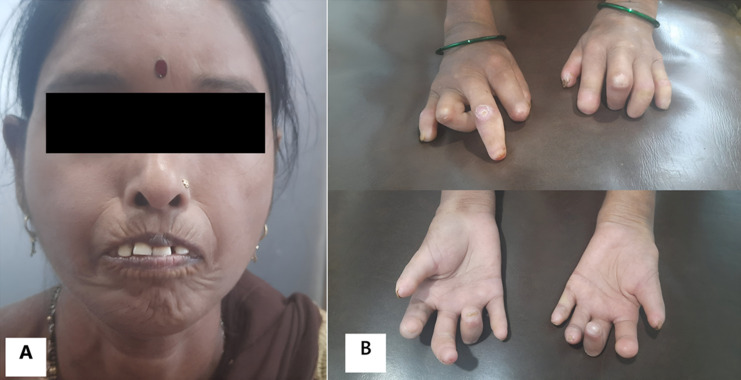
A) mask-like face with microstomia and prominent radial furrowing; B) dorsal and palmar aspects of the hand with acro-osteolysis, sclerodactyly, onycholysis, digital ulcers, and multiple finger flexion contractures

